# Assist-as-Needed Control for Upper Limb Rehabilitation Using a Learned Passive Interaction Force Reference

**DOI:** 10.3390/biomimetics11100765

**Published:** 2026-10-21

**Authors:** Xuanhan Li, Qiang Cao

**Affiliations:** 1 School of Mechatronic Engineering, China University of Mining and Technology, Xuzhou 221116, China; 2 School of Intelligent Manufacturing and Robotics, Shanghai DianJi University, Shanghai 201306, China; m15210819370@163.com

**Keywords:** upper limb rehabilitation robot, assist-as-needed control, passive interaction force, gated recurrent unit, participation estimation, adaptive impedance control

## Abstract

Upper-limb rehabilitation robots should provide adaptive assistance while avoiding excessive robotic intervention when users can contribute to movement. A key challenge is that measured interaction forces contain both voluntary and passive mechanical components, making direct force based participation assessment unreliable. This study proposes an Assist-as-Needed control framework based on a learned passive interaction force reference for a four degree of freedom upper-limb rehabilitation robot. Passive experiments with five healthy participants were conducted during eating and drinking tasks, and interaction forces were recorded at the upper-arm and forearm interfaces. The results revealed clear task, interface, and directional dependence in passive interaction. A Gated Recurrent Unit (GRU) model was then developed to estimate the expected passive interaction force from robot kinematics, movement phase, and task information, achieving a maximum (R^2^) of approximately 0.92. The deviation from the estimated passive reference was used to construct a control-oriented participation estimate, which was combined with trajectory tracking performance to regulate adaptive impedance. Controller-level simulation showed that robotic assistance decreased with increasing prescribed interaction contribution and increased again when tracking performance deteriorated. These results support the feasibility of using a learned passive interaction reference for Assist-as-Needed regulation and provide a basis for subsequent active human validation of the participation estimator.

## 1. Introduction

Upper-limb motor impairment caused by stroke and other neurological disorders can substantially reduce the ability to perform activities of daily living [1,2,3,4,5,6]. Robot assisted rehabilitation provides a means of delivering intensive and repeatable training while recording movement and interaction information during therapy [1,4,7,8,9,10,11]. Recent systematic reviews have continued to support the role of robotic systems in upper-limb rehabilitation, while also emphasizing that rehabilitation outcome depends not only on the amount of training but also on how robotic assistance is adapted to the user [1,2,3,4,5,6,11]. Conventional passive training can guide the limb along a prescribed trajectory, but it provides limited opportunity for the user to contribute to the movement [12,13,14]. Assist-as-Needed control addresses this issue by reducing robotic intervention when the user can perform the task and restoring assistance when additional support is required [15,16,17,18,19,20,21]. This principle has become an important direction in rehabilitation robotics because it aims to maintain task completion while encouraging active participation [15,21,22,23].

A central problem in Assist-as-Needed rehabilitation is how to determine the amount of assistance that should be provided at each moment [15,16,17,18,19,22]. Existing methods assess user performance from trajectory error, interaction force, electromyography, electroencephalography, physiological signals, or combinations of these variables [22]. Among them, interaction force is particularly attractive because it directly reflects the physical coupling between the user and the rehabilitation robot and can be measured continuously during training [23,24]. Force information has therefore been used to estimate motor capability, movement intention, and the required level of robotic support. Nevertheless, the interaction force measured at a human–robot interface is not equivalent to voluntary user effort. It also contains passive effects associated with limb dynamics, gravity, soft tissue deformation, attachment conditions, robot motion, and residual kinematic mismatch [25,26,27,28,29]. Directly mapping the measured interaction force to user participation may, therefore, cause passive mechanical effects to be interpreted as active contribution [15,22,23,24].

This problem is especially relevant for upper-limb rehabilitation robots because the human arm and robotic mechanism form a coupled kinematic system [8,9,30,31]. Anatomical joint centers change during movement, while the robot joints follow predefined mechanical axes [31]. Even when passive degrees of freedom are introduced to improve kinematic compatibility, interaction forces remain dependent on limb configuration and movement state [30]. Previous work on upper-limb rehabilitation robots has shown that human–robot interaction forces are strongly affected by robot structure, human limb dynamics, and kinematic compatibility, and accurate characterization of these forces is important for both comfort evaluation and interaction control [27,28,29,32,33]. Consequently, a fixed zero force assumption or a constant force threshold may not provide an appropriate reference for evaluating user contribution over a complete rehabilitation movement [15,22,24].

Data-driven methods provide another way to describe the changing interaction state without requiring a complete analytical model of the coupled human–robot system [23,24,34,35,36,37]. Machine learning has been increasingly applied to upper-limb rehabilitation for movement intention recognition, force estimation, human–robot interaction control, and motor function assessment. Recent studies have also used recurrent and other temporal neural networks to estimate interaction forces from kinematic or physiological signals because force generation during continuous movement depends on both the current state and previous motion history [24,36,37]. However, many force based rehabilitation methods either estimate voluntary force directly, rely on a robot or biomechanical dynamic model, or interpret measured interaction force as an indicator of user capability [25,26,27]. Comparatively less attention has been given to learning the expected passive interaction state itself and using the deviation from this state as the basis for participation estimation [15,22,23]. Such a reference may be particularly useful when passive interaction varies with movement phase, rehabilitation task, and physical interface.

Based on this consideration, this study develops an Assist-as-Needed control framework for a four degree of freedom (DOF) upper-limb rehabilitation robot using a learned passive interaction force reference. Passive rehabilitation experiments are first conducted during eating and drinking tasks, and interaction forces are measured simultaneously at the upper-arm and forearm interfaces. Released and locked passive joint conditions are compared to examine the effect of passive kinematic accommodation on the interaction load. The three dimensional interaction forces are then modeled using a Gated Recurrent Unit network with robot joint position, joint velocity, normalized movement phase, and task information as inputs. Rather than treating the estimated force as voluntary user effort, the network output represents the interaction state expected during passive robot-guided movement. The difference between the current measured force and this passive reference is projected along the desired movement direction and used to construct a normalized participation estimate. The upper-arm and forearm information is combined so that participation is evaluated from interaction changes observed at both physical interfaces. This design is consistent with the dual interface sensing architecture and passive interaction characterization established in the experimental part of the study.

The estimated participation level is finally combined with trajectory tracking performance to regulate an adaptive impedance controller [15,16,17,18,19,20,21,25,26,27]. This arrangement follows the basic principle of Assist-as-Needed rehabilitation but avoids reducing assistance solely because a larger interaction force is detected. Assistance is decreased only when increased interaction contribution is accompanied by acceptable movement performance, whereas robotic support can be restored when tracking deteriorates. The main contributions of this study are therefore threefold. First, passive interaction forces are characterized across rehabilitation tasks, physical interfaces, and passive joint conditions to establish the need for a task-dependent interaction reference. Second, a temporal GRU model is developed to predict task-dependent passive interaction forces at both physical interfaces, and the resulting directional residuals are used to construct a normalized participation estimate. Third, the resulting participation estimate is integrated with trajectory tracking performance to regulate robotic impedance within a unified Assist-as-Needed framework. Because active human subject data are not available in the present study, the final assistance regulation is evaluated through controller-level simulation, providing a controlled assessment of the proposed control logic before future active human and patient experiments.

## 2. Experimental Platform and Passive Interaction Force Characterization

### 2.1. Four DOF Upper-Limb Rehabilitation Robot and Sensing System

**Figure 1:**
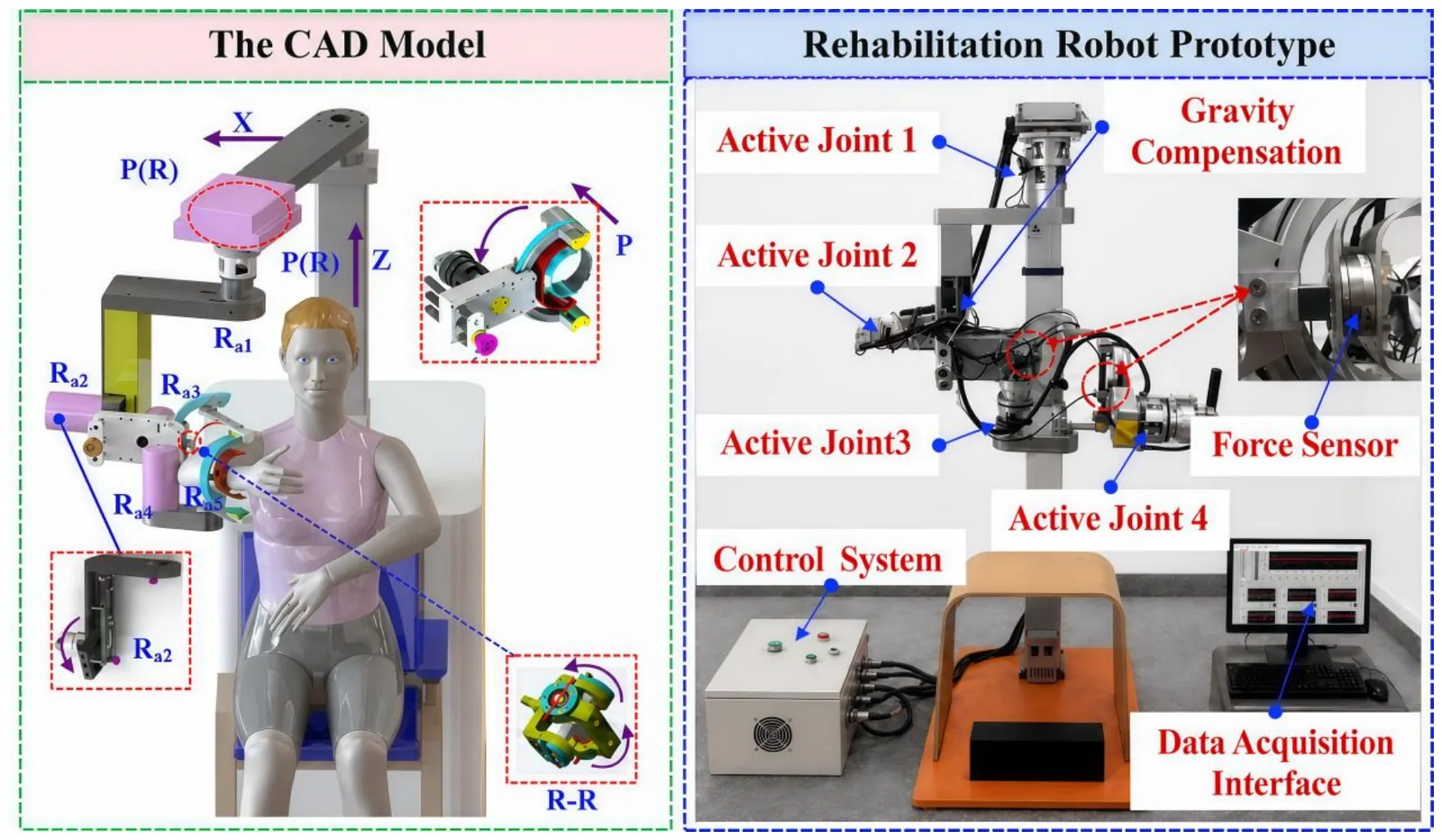
Four DOF upper-limb rehabilitation robot.

A four DOF upper-limb rehabilitation robot was used as the experimental platform in this study. The robot was developed to support coordinated rehabilitation training of the shoulder and elbow while maintaining physical compatibility with natural upper-limb motion. As illustrated in Figure 1, the robotic system consists mainly of an adjustable support structure, four actuated joints, passive adjustment joints, upper-arm and forearm interfaces, a gravity balancing mechanism, and the associated sensing and control system.

The four actuated joints correspond to shoulder abduction and adduction, shoulder flexion and extension, shoulder internal and external rotation, and elbow flexion and extension. The three rotational axes associated with the shoulder intersect approximately at a common point to reproduce the principal rotational characteristics of the human shoulder. In addition to the actuated joints, several passive translational and rotational joints are incorporated into the mechanical chain. These passive degrees of freedom accommodate variations in limb dimensions and the displacement of the anatomical joint centers during movement, thereby reducing unnecessary constraint forces arising from the closed kinematic interaction between the human arm and the robot. A spring based gravity balancing mechanism is also incorporated to compensate for part of the gravitational load of the robotic structure and reduce the actuator torque required during low speed rehabilitation movements.

Physical interaction between the participant and the robot is established through two attachment interfaces located at the upper arm and forearm. Two six-axis force–torque sensors are integrated into the interaction measurement system to record the forces generated at these interfaces. Joint positions are measured using incremental encoders mounted on the actuated joints. These measurements provide the joint motion and interaction force signals required for characterizing the physical coupling between the human arm and the rehabilitation robot.

The control system adopts a hierarchical architecture consisting of a host computer, a real-time embedded controller, four servo drives, joint encoders, force–torque sensors, and safety limit switches. The host computer is responsible for task selection, trajectory management, and experimental data recording, whereas the embedded controller executes joint motion commands and communicates with the servo drives through the CANopen protocol. During passive rehabilitation, the robot operates in position control mode and guides the participant along predefined rehabilitation trajectories. The synchronized joint motion and interaction force measurements obtained under this condition form the experimental basis for the passive interaction force modeling described in the following sections. To evaluate the physical interaction characteristics of the robot during representative functional upper-limb movements, two activities of daily living requiring coordinated shoulder and elbow motion and distinct upper-limb configurations, eating and drinking, were selected as the experimental tasks.

### 2.2. Activities of Daily Living Tasks and Data Acquisition

**Figure 2:**
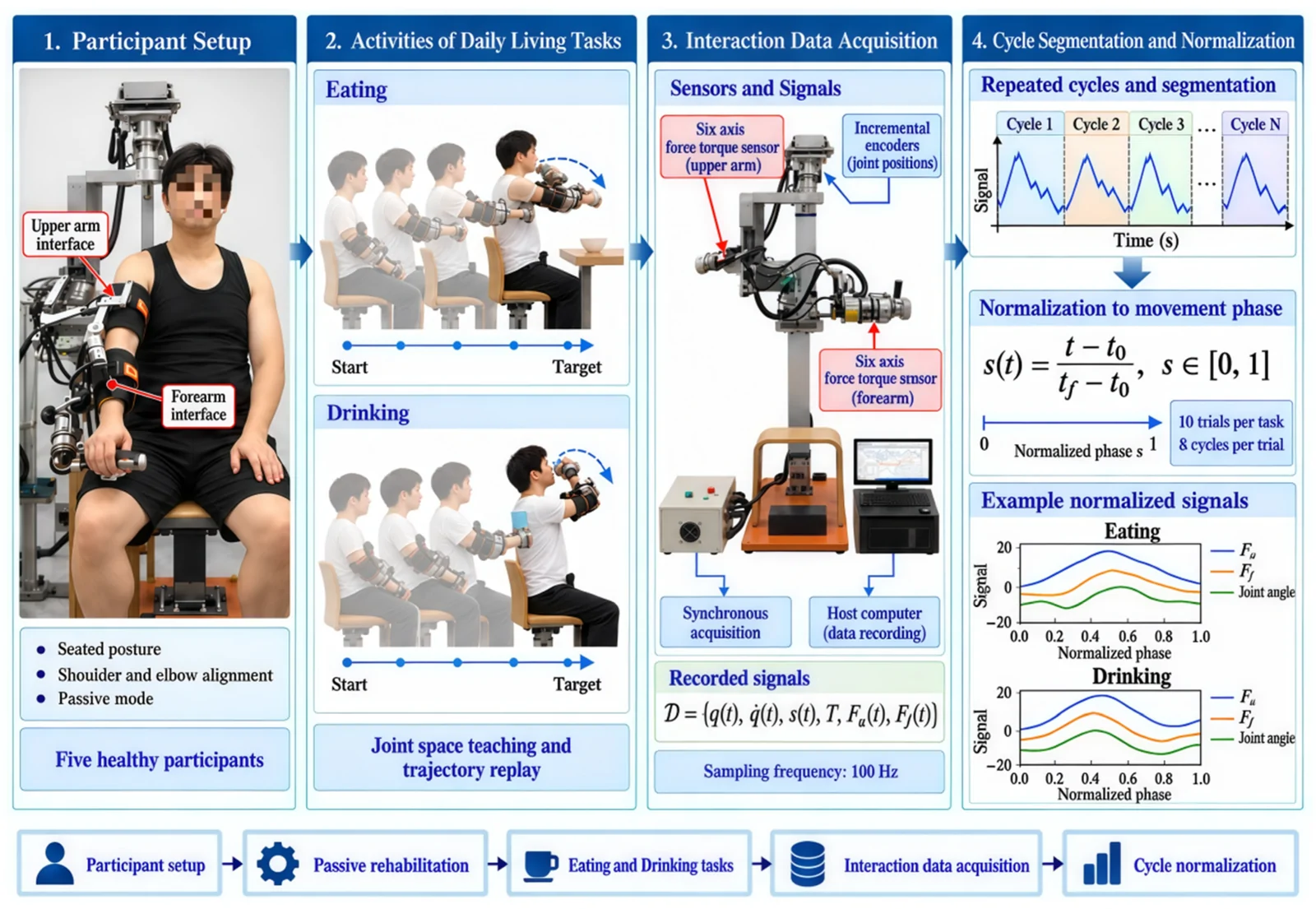
Experimental procedure and data acquisition for activities of daily living tasks.

Passive rehabilitation experiments were conducted to acquire human–robot interaction data under fully robot-guided motion. Five healthy participants with no history of neurological or musculoskeletal disorders affecting the upper limbs were recruited. The experimental protocol was approved by the Ethics Committee of Fuwai Hospital (approval No. 2022-1138) and was conducted in accordance with the Declaration of Helsinki and its later amendments. Written informed consent was obtained from all participants before participation in the experiments. Prior to data collection, the anthropometric dimensions of each participant were measured to adjust the height of the rehabilitation robot and the positions of the upper-arm and forearm interfaces. Participants were seated with the trunk supported, and the anatomical shoulder and elbow joints were aligned as closely as possible with the corresponding robot joints. The attachment interfaces were tightened to maintain stable physical contact without causing excessive compression. Several familiarization movements were performed before formal testing. During all experiments, participants were instructed to keep the right upper limb relaxed and to avoid voluntary contribution to the prescribed motion. The overall experimental procedure, including participant setup, execution of the two activities of daily living, synchronized acquisition of joint motion and interaction force signals, and subsequent cycle segmentation and normalization, is summarized in Figure 2.

The two tasks were performed under both released and locked passive joint conditions. In the released condition, the passive joints were allowed to move freely to accommodate joint center variations and reduce kinematic constraints within the human–robot closed chain. In the locked condition, the passive joints were mechanically constrained, thereby suppressing passive compensation and increasing the direct kinematic coupling between the participant and the rehabilitation robot. The same reference trajectories and data acquisition procedure were used for both conditions to enable direct comparison of the resulting interaction forces. Both tasks involve coordinated motion of the shoulder and elbow and produce substantial variations in upper-limb configuration during the movement. The corresponding joint trajectories were predefined through direct teaching in joint space and reproduced by the rehabilitation robot under position control. During each trial, the robot guided the participant along the prescribed trajectory using the four actuated joints. Joint positions were continuously measured using incremental encoders. To enable comparison among different repetitions and participants, each complete movement cycle was represented using a normalized movement phase

(1) $$s(t)=\frac{t-t_{0}}{t_{f}-{\;t}_{0}}$$

where *t*_0_ and *t_f_* denote the beginning and end of an individual movement cycle, respectively. Therefore,

*s*(*t*) ∈ [0, 1], (2)

with *s* = 0 representing movement initiation and *s* = 1 representing movement completion.

Human–robot interaction forces were simultaneously measured at the upper-arm and forearm interfaces using two identical six-axis force–torque sensors (Mini45, ATI Industrial Automation, Apex, NC, USA). One sensor was mounted between the upper-arm interface and the corresponding robot link, while the second sensor was installed at the forearm interface. Each sensor measured three orthogonal force components and three moment components. The present study primarily focuses on the interaction force signals measured at the two physical interfaces, denoted as *F_u_*(*t*) and *F_f_*(*t*), representing the upper-arm and forearm interaction forces, respectively. Joint motion and interaction force signals were synchronously recorded at a sampling frequency of 100 Hz.

Data acquisition started after a stable physical coupling state between the participant and the robot had been established. Each participant completed ten trials for both the eating and drinking tasks, and each trial consisted of eight continuous movement cycles. Identical reference trajectories and control parameters were maintained throughout repeated experiments. Mechanical joint limits, software constraints on joint position and velocity, an emergency stop mechanism, and continuous monitoring of interaction forces were used to ensure experimental safety. A trial was immediately terminated if a predefined safety condition was violated or if the participant reported discomfort.

After acquisition, the interaction force and joint motion signals were synchronized and segmented according to individual movement cycles. Each cycle was subsequently normalized with respect to movement phase. The resulting experimental dataset can be expressed as

(3) $$D=\left\{q\left(t\right),\dot{q}\left(t\right),s\left(t\right),F_{u}\left(t\right),F_{f}\left(t\right),T\right\},$$

where *q*(*t*) and $\dot{q}\left(t\right)$ denote the joint position and joint velocity vectors, respectively, *s*(*t*) represents the normalized movement phase, and *T* identifies the rehabilitation task. This dataset provides the experimental basis for the characterization and estimation of passive interaction forces presented in the following sections.

### 2.3. Characterization of Passive Interaction Forces

During passive rehabilitation, the prescribed movement was generated entirely by the rehabilitation robot while the participants were instructed to keep their upper limbs relaxed. Under this condition, the measured interaction forces primarily characterize the physical coupling between the human upper limb and the robot during the prescribed motion. These forces are influenced by the configuration and motion of the upper limb, gravitational and inertial effects, soft tissue deformation at the attachment interfaces, and residual kinematic mismatch between the anatomical and robotic joints [16,23,24,25,26,27,33]. Therefore, the measured signals were regarded as an empirical representation of the passive interaction state rather than as a direct measurement of passive muscle force. This distinction is important because the passive interaction state provides the reference condition used for the subsequent estimation of changes in human–robot interaction during assisted rehabilitation.

For each movement cycle, the three orthogonal force components measured at the upper-arm and forearm interfaces were expressed as

(4) $$F_{k}(t)=\left[\begin{array}{c} F_{x,k}\left(t\right)\\ F_{y,k}\left(t\right)\\ F_{z,k}\left(t\right) \end{array}\right],\;k\in \{u,\;f\}$$

where *u* and *f* denote the upper-arm and forearm interfaces, respectively. The corresponding resultant interaction force was calculated as

(5) $$F_{\mathrm{total},k}(t)={\left\|F_{k}\left(t\right)\right\|}_{2}=\sqrt{F_{x,k}^{2}\left(t\right)+F_{y,k}^{2}\left(t\right)+F_{z,k}^{2}\left(t\right)}.$$

The three dimensional force vectors were retained to preserve directional interaction information for subsequent modeling, whereas the resultant force was used as a compact measure for comparing the overall interaction intensity between tasks and physical interfaces.

To account for differences in movement duration among repetitions, the force signals of each movement cycle were mapped from the time domain to the normalized movement phase ss defined in Section 2.2. Each cycle was represented using the same uniformly distributed phase samples over *s* ∈ [0, 1]. For participant *i*, task *T*, and movement cycle *c*, the normalized interaction force can, therefore, be represented as

(6) $$F_{k,i,c}^{\left(T\right)}\left(s\right),\;T\in \left\{\mathrm{eating},\mathrm{drinking}\right\}.$$

A representative passive interaction force profile for each participant was obtained by averaging the repeated movement cycles,

(7) $${\bar{F}}_{k,i}^{\left(T\right)}(s)=\frac{1}{C}\sum_{c=1}^{C}F_{k,i,c}^{\left(T\right)}(s),$$

where *C* denotes the number of valid movement cycles. Group level profiles were subsequently obtained from the participant specific profiles, allowing the variation in interaction forces throughout the movement cycle to be examined without assigning excessive weight to individual repetitions.

The passive interaction force data were characterized from three complementary aspects. First, variation in the force profile over the normalized movement phase was examined to determine whether the physical interaction exhibited consistent temporal characteristics during a rehabilitation movement. Second, the force profiles obtained during eating and drinking were compared to assess the influence of task-specific upper-limb motion on the interaction state. Third, the measurements from the upper-arm and forearm interfaces were examined separately to determine whether the two physical interfaces contained complementary interaction information. Mean force, peak force, and the corresponding movement phase were used as descriptive measures of the interaction characteristics.

The averaged profiles were used only for descriptive characterization and comparison of the passive interaction state. The individual movement cycles and their associated joint motion signals were retained as independent sequences for the passive interaction force estimation developed in Section 3.1. In this way, the temporal variation and trial variability contained in the original measurements were preserved for data-driven modeling.

## 3. Passive Interaction Force-Guided Assist-as-Needed Control

### 3.1. Passive Interaction Force Estimation Using a GRU Network

The passive interaction force measured during robot-guided rehabilitation varies continuously with the configuration and motion state of the human–robot system. As described in Section 2.3, the force profiles also depend on the movement phase, rehabilitation task, and physical interaction interface. Therefore, estimating the passive interaction force solely from the instantaneous joint configuration may neglect the temporal dependence associated with continuous upper-limb movement. A GRU network was consequently introduced to establish the mapping between robot kinematics and the interaction forces measured at the upper-arm and forearm interfaces. Since the released passive joint configuration represents the intended operating condition for subsequent Assist-as-Needed rehabilitation, only data acquired under the released condition were used for training and evaluating the passive interaction force estimator. The locked condition was retained solely for evaluating the influence of passive kinematic compensation on human–robot interaction in Section 4.1. Recurrent neural networks have been increasingly used for force estimation because their internal states allow information from previous time steps to contribute to the current prediction, which is particularly suitable for continuous physical human–robot interaction [30,33,36,37].

At each sampling instant *t*, the model input consists of the joint positions, joint velocities, normalized movement phase, and rehabilitation task information defined in Section 2.2. The input vector is expressed as

(8) $$x_{t}={\left[\begin{array}{cccc} q_{t}^{T} & {\dot{q}}_{t}^{T} & s_{t} & c_{T}^{T} \end{array}\right]}^{T}\in R^{11}$$

where *q_t_* = [*q*_1,*t*_, *q*_2,*t*_, *q*_3,*t*_, *q*_4,*t*_]*^T^* and $\dot{q}(t)\;={\;[\begin{array}{cc} {\dot{q}}_{1,t} & {\dot{q}}_{2,t} \end{array}\begin{array}{cc} & \begin{array}{cc} {\dot{q}}_{3,t} & {\dot{q}}_{4,t} \end{array} \end{array}]}^{T}$ denote the angular position and angular velocity vectors of the four actuated joints, respectively. The *s_t_* denotes the normalized movement phase defined in Equation (1), and *c_T_* is a binary categorical vector representing the rehabilitation task. The eating and drinking are encoded as [1, 0]*^T^* and [0, 1]*^T^*, respectively. Therefore, the input dimension of the GRU estimator is 11. Participant specific anthropometric measurements were used to adjust the robot configuration and the positions of the upper-arm and forearm interfaces during participant setup, but these anthropometric variables were not explicitly included as input features of the present GRU estimator.

To preserve the temporal information contained in the rehabilitation movement, consecutive input vectors within a finite observation window are organized as an input sequence

(9) $$X_{t}={\left[\begin{array}{cccc} x_{t-L+1} & \cdots & x_{t-1} & x_{t} \end{array}\right]}^{T},$$

where *L* denotes the sequence length. The prediction target consists of the three dimensional interaction forces measured at both physical interfaces,

(10) $$y_{t}={\left[\begin{array}{cc} (F_{u,t}^{T}{)}^{T} & (F_{f,t}^{T}{)}^{T} \end{array}\right]}^{T}\in R^{6}.$$

Thus, the estimation problem can be formulated as

(11) $${\hat{y}}_{t}=G\left(X_{t};\theta \right)$$

where *G*(.) denotes the GRU model and ***θ*** represents the trainable model parameters. The model simultaneously estimates the interaction force vectors at the upper-arm and forearm interfaces,

(12) $${\hat{y}}_{t}={\left[\begin{array}{cc} ({\hat{F}}_{u,t}^{T}{)}^{T} & ({\hat{F}}_{f,t}^{T}{)}^{T} \end{array}\right]}^{T}$$

The recurrent state of the network is updated through the reset and update gates. For an input vector *x_t_*, the corresponding operations are expressed as

(13) $$\begin{array}{rrr} r_{t} & = & \sigma \left(W_{r}x_{t}+U_{r}h_{t-1}+b_{r}\right),\\ z_{t} & = & \sigma \left(W_{z}x_{t}+U_{z}h_{t-1}+b_{z}\right),\\ {\tilde{h}}_{t} & = & \tanh\left[W_{h}x_{t}+U_{h}\left(r_{t}⊙h_{t-1}\right)+b_{h}\right],\\ h_{t} & = & \left(1-z_{t}\right)⊙h_{t-1}+z_{t}⊙{\tilde{h}}_{t} \end{array},$$

where *r_t_* and *z_t_* denote the reset and update gates, respectively, h*_t_* is the hidden state, *σ*(.) is the sigmoid activation function, and ⊙ denotes elementwise multiplication. The estimated interaction force is obtained from the hidden state through a fully connected output layer,

(14) $${\hat{y}}_{t}=W_{o}h_{t}+b_{o}$$

The GRU estimator consisted of two recurrent layers followed by a fully connected output layer. The first and second GRU layers contained 64 and 32 hidden units, respectively. A dropout layer with a rate of 0.20 was introduced between the recurrent layers to reduce overfitting. The final hidden representation was mapped to a six dimensional output vector corresponding to the three interaction force components measured at the upper-arm and forearm interfaces. The sequence length was set to *L* = 50 samples. Since the interaction signals were recorded at 100 Hz, each input sequence represented 0.5 s of movement history. Consecutive temporal windows were generated within each individual movement cycle, and no sequence was allowed to cross the boundary between two adjacent cycles. This setting provides a finite temporal context for describing the continuous evolution of passive interaction forces while preserving local changes during the rehabilitation movement. The architecture and main training settings of the GRU estimator are summarized in Table 1.

Before model training, the kinematic and force variables were standardized separately for each cross-validation fold. The normalization parameters were calculated exclusively from the training data and were subsequently applied without modification to the corresponding validation and test data. Complete movement cycles were retained as independent temporal sequences, and all temporal windows generated from the same cycle were assigned to the same data subset to prevent information leakage between model development and evaluation.

Considering the limited number of participants, model generalization was evaluated using fivefold leave-one-participant-out cross-validation. In each fold, all movement cycles from one participant were held out as an independent test set, while data from the remaining four participants were used for model development. This procedure was repeated five times so that each participant served once as the unseen test participant. Therefore, no movement cycle or sampling point from the test participant was included during model training or validation.

Within the four participants used for model development, complete movement cycles were divided into training and validation subsets at a ratio of 80:20. All temporal windows originating from the same movement cycle were retained in the same subset. The GRU model was initialized and trained independently for each outer fold using the same network architecture and hyperparameters listed in Table 1. This procedure was designed to evaluate the ability of the estimator to predict passive interaction forces for a participant who was not represented during model training.

The network parameters are optimized by minimizing the mean squared estimation error between the measured and estimated interaction forces,

(15) $$L=\frac{1}{N}\sum_{t=1}^{N}{\left\|y_{t}-{\hat{y}}_{t}\right\|}_{2}^{2},$$

where *N* denotes the number of samples used for model optimization. The network was trained using the Adam optimizer with an initial learning rate of 1 × 10^−3^, a batch size of 32 sequences, and a maximum of 200 epochs. An *L*_2_ weight decay of 1 × 10^−5^ was applied during optimization. Early stopping was employed when the validation loss did not improve for 20 consecutive epochs, and the model parameters corresponding to the lowest validation loss were retained for testing. The same training settings were used for all five cross-validation folds.

Model performance was evaluated separately for each held out participant using the root mean square error (RMSE), mean absolute error (MAE), and coefficient of determination (R^2^). Participant independent performance was subsequently summarized across the five outer folds. For quantitative comparison, Linear Regression (LR) and a Multilayer Perceptron (MLP) were employed as reference models in addition to the proposed GRU estimator. The three models used the same kinematic, movement phase, and task information as input. Their prediction performance was evaluated at both the upper-arm and forearm interfaces under the eating and drinking tasks. To provide a concise task-specific comparison, the component averaged RMSE and coefficient of determination (R^2^) across *F_x_*, *F_y_*, *F_z_* were used as summary indicators. In addition, RMSE, MAE, and R^2^ were calculated separately for each force component to provide a detailed evaluation of the six-dimensional force estimation performance. The corresponding quantitative comparison is presented in Section 4.2.

Importantly, the estimated forces ${\hat{F}}_{u,t}$ and ${\hat{F}}_{f,t}$ represent the expected interaction state under passive robot-guided movement rather than voluntary forces generated by the participant. They therefore provide a data-based passive interaction reference for the subsequent calculation of interaction force residuals and participation estimation described in Section 3.2.

### 3.2. Participation Estimation from Interaction Force Residuals

**Figure 3:**
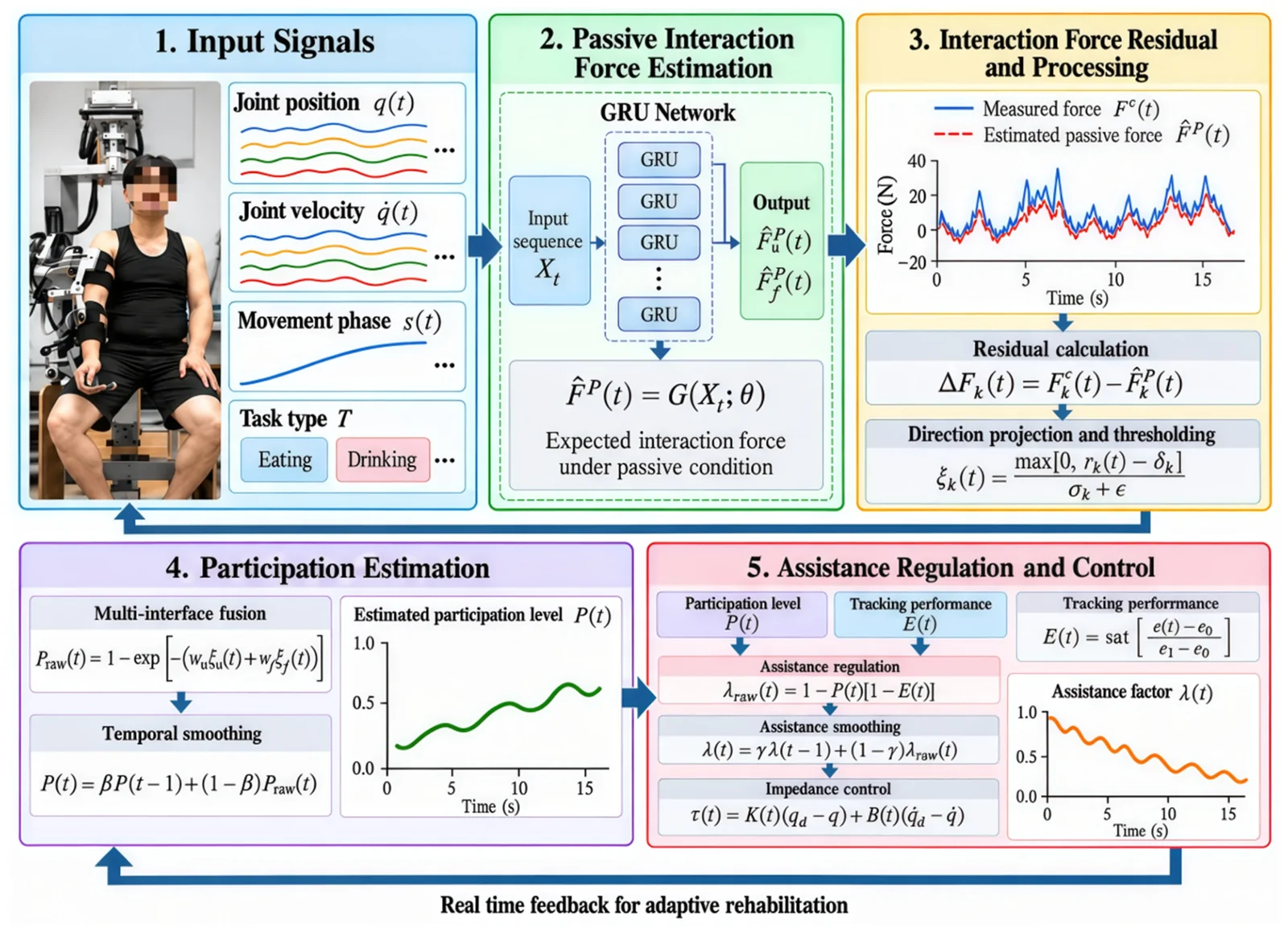
Overall framework of interaction force-based participation estimation and control.

The passive interaction force model established in Section 3.1 provides the expected interaction state during robot-guided movement without voluntary contribution. During assisted movement, deviations from this reference may arise when the current human–robot interaction changes. The present section therefore quantifies these deviations in the prescribed movement direction and converts them into a normalized control-oriented participation estimate. The overall information flow is illustrated in Figure 3.

For each physical interface *k* ∈ {*u*, *f*}, the interaction force residual is defined as

(16) $$\Delta F_{k}(t)=F_{k}^{c}(t)\;-{\hat{\;F}}_{k}^{p}(t),$$

where $F_{k}^{c}\left(t\right)$ denotes the current interaction force at the upper-arm or forearm interface, and ${\hat{F}}_{k}^{p}\left(t\right)$ is the corresponding passive interaction force estimated by the GRU model. All force vectors are expressed in a common robot coordinate frame before the residual is calculated. Under an ideal passive condition, Δ*F_k_(t)* approaches zero. A systematic deviation from the passive reference therefore indicates a change in the physical contribution of the user to the human–robot interaction.

The magnitude of the force residual alone, however, cannot distinguish whether the additional interaction assists or opposes the prescribed motion. To retain this directional information, the residual force is projected onto the instantaneous direction of the desired movement at each attachment interface. Let $\hat{v_{k}}\left(t\right)$ denote the unit vector along the desired interface motion. The directional interaction residual is defined as

(17) $$r_{k}(t)=\Delta F_{k}^{T}\left(t\right){\hat{v}}_{k}\left(t\right),$$

where the sign convention is selected such that a positive value corresponds to an interaction contribution in the prescribed movement direction. Consequently, a positive residual indicates interaction that is consistent with the desired movement, whereas a negative residual represents interaction opposing the movement. This directional projection prevents increases in interaction force caused by resistance or kinematic mismatch from being incorrectly interpreted as increased participation.

Because the passive force estimator has finite prediction uncertainty, small residuals may be present even when no additional interaction contribution occurs. An interface specific residual tolerance, *δ_k_*, was therefore determined from the passive validation residuals. Specifically, *δ_k_* was defined as the 95th percentile of the absolute directional residual obtained under the released passive condition. This threshold excludes most residual fluctuations attributable to estimation uncertainty and measurement noise while retaining larger deviations for participation estimation. Only the residual component exceeding this passive uncertainty range was retained,

(18) $$\xi_{k}(t)=\frac{\max\left[0,{\;r}_{k}\left(t\right)-\delta_{k}\right]}{\sigma_{k}+\varepsilon },$$

where *σ_k_* denotes the standard deviation of the corresponding passive directional residual at interface *k*, and *ε* is a small positive constant introduced to avoid numerical singularity. The use of an interface specific *σ_k_* compensates for the different force ranges and residual variability observed at the upper-arm and forearm interfaces. This formulation therefore provides a normalized measure of deviation from the expected passive interaction state while reducing sensitivity to sensor noise and passive model uncertainty.

The upper-arm and forearm interfaces provide interaction information from different segments of the human upper limb. Their normalized residual measures are therefore combined to obtain a single control-oriented participation estimate,

*P*_raw_(*t*) = 1 − *exp* [−(*w_u_ξ_u_*(*t*) + *w_f_ξ_f_*(*t*))]. (19)

Because the residual measures at the two physical interfaces were normalized by their respective passive residual variability before fusion, equal interface weights were adopted in the present study, with *w_u_* = *w_f_* = 0.50. This setting avoids introducing an a priori preference for either interface and is consistent with the experimental observation that the upper arm and forearm provide complementary interaction information whose relative contribution varies between eating and drinking. The same interface weights were used for all participants and tasks.

*w_u_* + *w_f_* = 1, *w_u_* = *w_f_* = 0.50. (20)

The exponential mapping constrains the estimated participation level to the interval

*P*_raw_(*t*) ∈ [0, 1), (21)

while preserving a monotonic relationship between the interaction force deviation and the resulting participation estimate. To suppress rapid fluctuations caused by measurement noise and transient contact variations, the raw estimate is subsequently smoothed using

*P*(*t*) = *βP*(*t* − 1) + (1 − *β*)*P*_raw_(*t*), (22)

where 0 < *β* < 1 determines the degree of temporal smoothing. In the present implementation, the participation smoothing coefficient was set to *β* = 0.90. Considering the 100 Hz sampling frequency, this value was selected to suppress sample level fluctuations caused by force sensing noise and transient contact variations while retaining the slower movement-related changes required for assistance regulation. The same value was used for all participants and rehabilitation tasks.

The resulting *P*(*t*) is referred to as the estimated participation level in the remainder of this study. A value close to zero indicates that the current physical interaction remains close to the passive reference, whereas an increase in *P*(*t*) indicates a progressively stronger interaction contribution in the prescribed movement direction. Importantly, *P*(*t*) is introduced as a control variable derived from deviations in human–robot interaction and should not be interpreted as a direct measurement of muscle force, neural activation, or clinical motor ability. The estimated participation level is subsequently combined with trajectory tracking performance to regulate the amount of robotic assistance in Section 3.3. In the present study, *P*(*t*) has not been experimentally calibrated or validated against voluntary motor effort; therefore, it should be interpreted as a control-oriented interaction based participation estimate rather than a physiological measure of voluntary contribution.

### 3.3. Assistance Regulation and Adaptive Impedance Control

The estimated participation level *P*(*t*) obtained in Section 3.2 characterizes the extent to which the current human–robot interaction deviates from the learned passive interaction state in the prescribed movement direction. However, participation information alone is insufficient to determine the appropriate amount of robotic assistance [17,18,19,20,21,22]. A user may exhibit a relatively high interaction contribution while failing to follow the desired rehabilitation trajectory accurately. Conversely, a small tracking error may result from strong robotic guidance rather than sufficient user contribution. Therefore, the proposed Assist-as-Needed strategy jointly considers participation and trajectory tracking performance when regulating robotic assistance.

Since the eating and drinking trajectories are defined in joint space, the instantaneous tracking error is evaluated from the four actuated joints. The joint error vector and its normalized scalar measure are defined as

(23) $$\begin{array}{rrr} e_{q}\left(t\right) & = & q_{d}\left(t\right)-q(t),\\ e_{n}\left(t\right) & = & \sqrt{\frac{1}{4}e_{q}^{T}\left(t\right){\;W}_{e}e_{q}\left(t\right)},\\ E(t) & = & {\mathrm{sat}}_{\left[0,1\right]}\left[\frac{e_{n}\left(t\right)-e_{0}}{e_{1\;}-e_{0}}\right] \end{array},$$

where *q_d_*(*t*) and *q*(*t*) denote the desired and measured joint position vectors, respectively. To account for the different motion ranges of the four actuated joints, the diagonal weighting matrix was defined according to the corresponding trajectory ranges,

(24) $$W_{e}=\mathrm{diag}\left(\frac{1}{\Delta q_{1}^{2}},\frac{1}{\Delta q_{2}^{2}},\frac{1}{\Delta q_{3}^{2}},\frac{1}{\Delta q_{4}^{2}}\right),$$

where Δ*q_j_* denotes the motion range of joint *j* for the corresponding rehabilitation trajectory. The tracking error is therefore evaluated relative to the prescribed motion range rather than directly from the absolute error in radians.

The acceptable tracking threshold and upper correction boundary were set to *e*_0_ = 0.05 and *e*_1_ = 0.20, respectively. Tracking deviations below approximately 5% of the normalized joint motion range were treated as acceptable, whereas deviations reaching approximately 20% corresponded to the upper correction boundary. Errors between these two limits were mapped continuously to *E*(*t*) ∈ [0, 1]. These limits were selected as conservative controller-level bounds for distinguishing normal tracking variation from a clear deterioration in movement performance and should not be interpreted as clinically validated impairment thresholds.

The participation estimate and tracking performance are then integrated into a normalized assistance factor,

*λ*_raw_(*t*) = 1 − *P*(*t*)[1 − *E*(*t*)],  *λ*_raw_(*t*) ∈ [0, 1]. (25)

This regulation law reflects the fundamental principle of Assist-as-Needed rehabilitation. When the estimated participation level is low, *P*(*t*) approaches zero and the assistance factor remains close to unity, indicating that substantial robotic support is required. When the user exhibits a high interaction contribution and simultaneously maintains accurate trajectory tracking, *P*(*t*) increases while *E*(*t*) remains small, resulting in a reduction in robotic assistance. In contrast, an increase in tracking error raises *λ_raw_*(*t*), allowing robotic support to be restored even when a relatively high participation level is detected. In this way, reduced assistance is permitted only when the user contribution is accompanied by adequate movement performance.

To avoid abrupt variations in robotic behavior caused by transient force fluctuations or short-term tracking disturbances, the assistance factor is temporally smoothed as

*λ*(*t*) = *γλ*(*t* − 1) + (1 − *γ*)*λ*_raw_(*t*),  0 < *γ* < 1. (26)

The assistance smoothing coefficient was set to *γ* = 0.90. Consistent with the participation smoothing coefficient, this value was selected to suppress abrupt assistance changes caused by short term fluctuations while retaining sufficient responsiveness to deterioration in trajectory tracking. At the 100 Hz controller update rate, this setting produces a smooth transition between assistance levels without introducing an excessively slow controller response.

The resulting *λ*(*t*) is not directly interpreted as an actuator torque. Instead, it regulates the mechanical impedance of the rehabilitation robot. The joint stiffness and damping matrices are varied continuously between predefined lower and upper bounds according to

(27) $$\begin{array}{rrr} K\left(t\right) & = & K_{\min}+\lambda \left(t\right)\left(K_{\max}-K_{\min}\right)\\ B\left(t\right) & = & B_{\min}+\lambda \left(t\right)\left(B_{\max}-B_{\min}\right) \end{array}.$$

where *K_min_*, *K_max_*, *B_min_*, and *B_max_* are positive diagonal matrices defining the allowable impedance range of the four actuated joints. The corresponding assistive joint torque is given by

(28) $$\tau_{a}\left(t)=K(t\right)e_{q}\left(t)+B(t\right)\left[{\dot{q}}_{d}\left(t\right)-\dot{q}\left(t\right)\right].$$

The commanded torque is restricted within the allowable torque range of each actuator to ensure that the generated assistance remains within the predefined safety limits. This formulation enables a continuous transition between robot dominant and user dominant rehabilitation. When participation is limited or movement performance deteriorates, the assistance factor increases and the robot provides stronger trajectory guidance through increased virtual stiffness and damping. When the user contributes effectively while maintaining acceptable tracking performance, the assistance factor decreases, resulting in a more compliant interaction and reduced robotic intervention. The measured interaction force therefore influences robotic impedance only through the estimated participation level and the subsequent assistance regulation, rather than being mapped directly to actuator torque. This hierarchical regulation completes the closed loop Assist-as-Needed control framework illustrated in Figure 3.

Overall, the main control parameters were selected according to two complementary principles. Parameters associated with passive interaction uncertainty, including *δ_k_* and *σ_k_*, were derived directly from the passive validation residuals and were therefore interface-dependent. In contrast, the interface weights, temporal smoothing coefficients, and tracking error limits were treated as fixed controller design parameters and were kept identical across participants and rehabilitation tasks. Specifically, *w_u_* = *w_f_* = 0.50, *β* = *γ* = 0.90, and *e*_0_ = 0.05 and *e*_1_ = 0.20. These settings provide balanced dual interface–information fusion, suppress transient measurement fluctuations, and allow a continuous transition from acceptable trajectory tracking to assistance restoration. No participant-specific parameter tuning was performed.

## 4. Results and Discussion

### 4.1. Passive Interaction Force Characteristics

**Figure 4:**
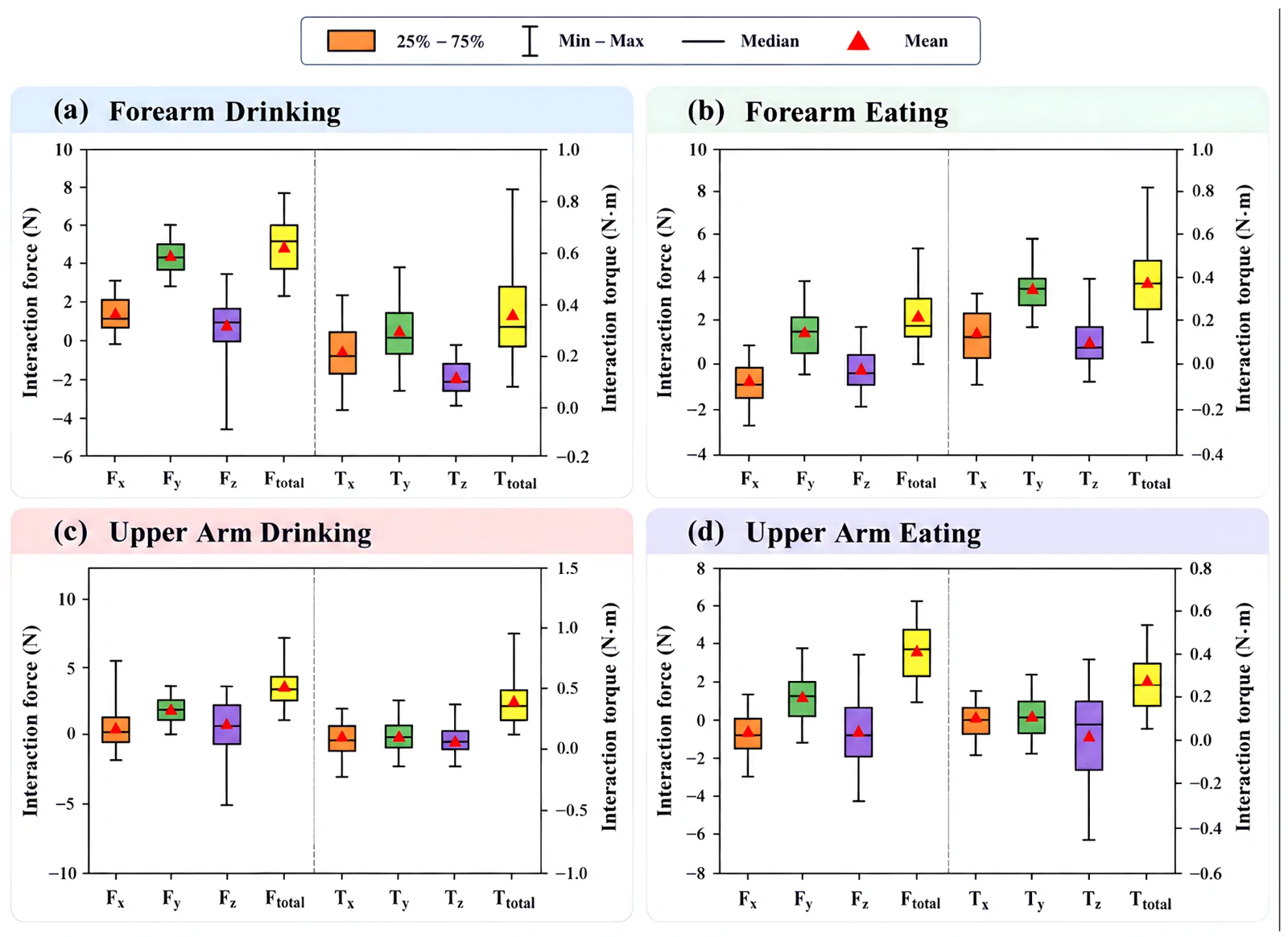
Statistical distributions of passive interaction forces across tasks and interfaces.

The statistical distributions of the passive interaction forces and torques measured during the eating and drinking tasks are presented in Figure 4. Considerable differences were observed across rehabilitation tasks, physical interfaces, and force directions. In particular, the resultant interaction force and the individual Cartesian force components did not vary proportionally across conditions, indicating that passive human–robot interaction cannot be represented adequately by a single constant load. Instead, the interaction state exhibited clear task-dependent, interface-dependent, and directional characteristics.

At the forearm interface, the drinking task produced a markedly greater resultant interaction force than the eating task. At the forearm interface, the mean resultant force increased from 2.69 N under the released condition to 4.39 N under the locked condition during eating, and from 5.33 to 7.85 N during drinking. The consistently higher resultant force observed after locking the passive joints indicates that passive kinematic accommodation effectively reduces interaction loading at the human–robot interface.

This increase was accompanied by a pronounced contribution from the *F_y_* component, which remained one of the dominant positive force components in both tasks. The *F_z_* component, however, showed substantial variation in both magnitude and direction, suggesting that the loading state of the forearm interface changed with upper-limb configuration rather than simply scaling with the overall interaction magnitude.

The upper arm exhibited a different task related pattern. The corresponding mean resultant forces were 3.18 and 5.12 N during eating and 3.64 and 4.14 N during drinking. Unlike the forearm, the upper arm did not exhibit a consistent increase in resultant force during drinking. Instead, the individual directional components changed more noticeably. In particular, the *F_z_* component showed both positive and negative values depending on the task and experimental condition, whereas *F_y_* remained predominantly positive. These results indicate that the mechanical interaction at the upper arm is influenced strongly by the orientation and configuration of the shoulder rather than by the overall movement intensity alone.

**Figure 5:**
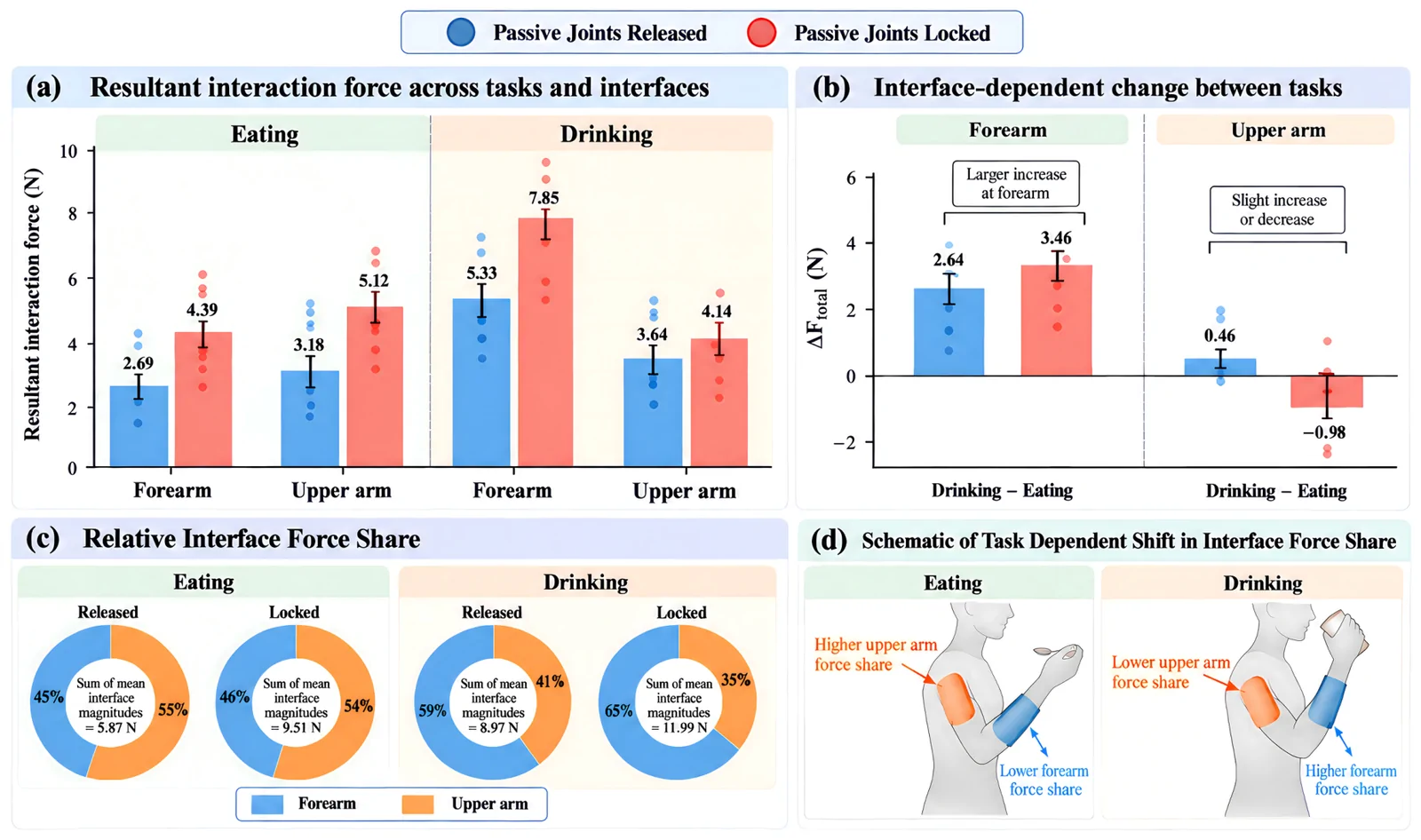
Task and interface dependence of the resultant passive interaction force. (**a**) Mean resultant interaction force at the forearm and upper-arm interfaces during eating and drinking under passive joints released and passive joints locked conditions; bars and error bars represent the mean ± SD, and dots indicate individual participants. (**b**) Task-dependent change in the resultant interaction force between drinking and eating at each interface. (**c**) Relative interface force share calculated by normalizing the mean resultant force magnitude at each interface by the sum of the forearm and upper-arm resultant force magnitudes. (**d**) Schematic illustration of the task-dependent shift in relative interface force share along the upper limb.

The influence of passive joint status on the task-dependent interaction pattern is further summarized in Figure 5. The two experimental conditions are denoted as passive joints released and passive joints locked, which describe the mechanical states of the passive joints rather than different levels of robotic assistance. For Figure 5c, the relative interface force share was calculated from the mean resultant interaction force magnitudes measured at the forearm and upper-arm interfaces. Specifically,

(29) $$S_{f}=\frac{{\bar{F}}_{\mathrm{total},f}}{{\bar{F}}_{\mathrm{total},f}+{\bar{F}}_{\mathrm{total},u}}\times 100\%,\;S_{u}=\frac{{\bar{F}}_{\mathrm{total},u}}{{\bar{F}}_{\mathrm{total},f}+{\bar{F}}_{\mathrm{total},u}}\times 100\%.$$

where *S_f_* and *S_u_* denote the relative force shares of the forearm and upper-arm interfaces, respectively, and ${\bar{F}}_{\mathrm{total},f}$ and ${\bar{F}}_{\mathrm{total},u}$ denote the corresponding mean resultant interaction force magnitudes. The denominator is used only for normalization and does not represent a vector summation of the two interface forces or the net mechanical load acting on the upper limb. Therefore, the term relative interface force share is used to describe the relative magnitudes measured at the two physical interfaces. Using this definition, the upper arm accounted for approximately 54.2% and 53.8% of the two interface force magnitudes during eating under the released and locked conditions, respectively. In contrast, during drinking, the forearm share increased to approximately 59.4% in the released condition and 65.5% in the locked condition. These results indicate a task-dependent shift in the relative interaction force measured at the two interfaces rather than a simple increase in the overall interaction magnitude.

To provide a quantitative summary of the passive interaction characteristics, the mean and standard deviation of the resultant interaction force across different rehabilitation tasks, physical interfaces, and passive joint conditions are summarized in Table 2. The results further demonstrate the dependence of passive interaction loading on both the physical interface and the mechanical state of passive joints.

The torque measurements showed a different behavior from the force measurements. As shown in Figure 4, the resultant interaction torques remained within a comparatively narrow range across tasks and interfaces. For example, the mean *T_total_* values ranged approximately from 0.18 to 0.45 N·m across the four task and interface combinations. Moreover, changes in resultant torque did not consistently follow the corresponding changes in resultant force. This suggests that the task-dependent variation was more pronounced in the transmitted force than in the overall interaction torque. Nevertheless, the directional torque components still exhibited different distributions across tasks and interfaces, providing complementary information on the local loading state.

Another notable feature was the difference between the resultant force and the directional force components. Several components, particularly *F_z_*, exhibited broad distributions and even changes in sign, despite relatively moderate changes in *F_total_*. Therefore, the resultant force alone cannot fully describe the interaction state because it removes information regarding the direction of load transmission. This observation supports the retention of the three dimensional interaction force vectors at both the upper-arm and forearm interfaces for subsequent passive force estimation.

Overall, the experimental results demonstrate that passive human–robot interaction is jointly influenced by rehabilitation task, physical interface, and force direction. The task-dependent shift in relative interface force share between the upper arm and forearm during eating and drinking further confirms that the two sensing interfaces provide complementary rather than redundant interaction information. These characteristics provide experimental support for incorporating task information and dual interface force measurements into the passive interaction force estimation model developed in Section 3.1.

### 4.2. Passive Interaction Force Estimation Performance

**Figure 6:**
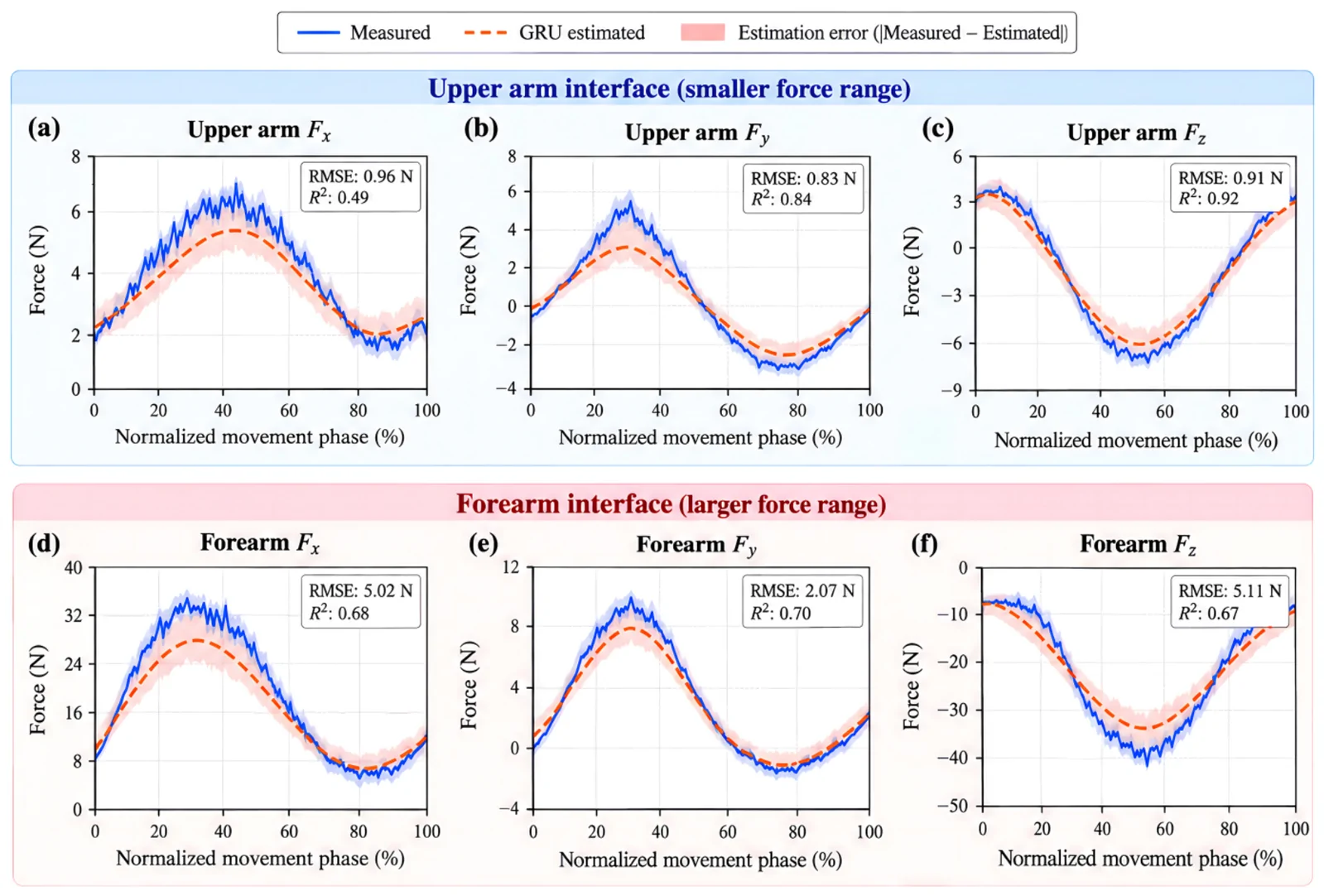
Measured and estimated passive interaction forces over a normalized movement cycle.

The passive interaction force estimation results are shown in Figure 6. The blue curves represent the measured interaction forces, while the red curves represent the forces estimated by the GRU model. The measured forces showed clear periodic changes during repeated rehabilitation movements. The predicted curves followed the main variation in the measured signals at both the upper-arm and forearm interfaces. The locations of the main peaks and valleys were also reproduced well.

**Figure 7:**
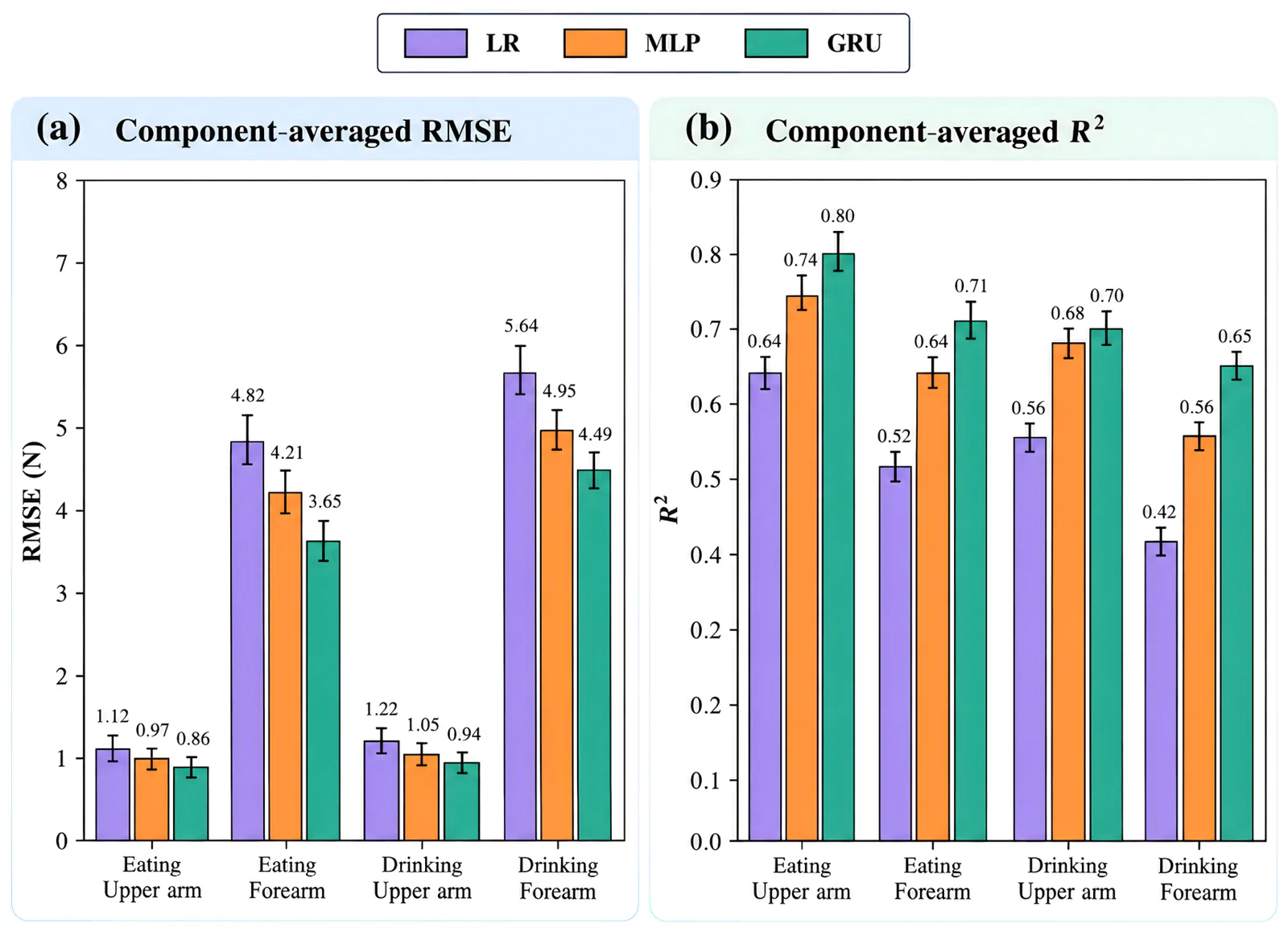
Task-specific quantitative comparison of passive interaction force estimation performance. (**a**) Component averaged RMSE and (**b**) component averaged R^2^ for LR, MLP, and GRU under the eating and drinking tasks at the upper-arm and forearm interfaces. The component averaged values were calculated from the corresponding performance of *F_x_*, *F_y_*, and *F_z_*.

In addition to the component-wise temporal comparison shown in Figure 6, a task-specific quantitative comparison among the LR, MLP, and GRU estimators is presented in Figure 7. To provide a compact representation of the overall estimation performance while retaining the distinction between rehabilitation tasks and physical interfaces, the RMSE and R^2^ values of *F_x_*, *F_y_*, and *F_z_* were averaged within each task and interface condition. Accordingly, the comparison was performed separately for eating and drinking at the upper-arm and forearm interfaces.

As shown in Figure 7, the GRU estimator generally provided better overall prediction performance than the LR and MLP reference models under both rehabilitation tasks. The GRU produced lower component averaged RMSE values together with higher component averaged R^2^ values across the upper-arm and forearm interfaces. The improvement from LR to MLP suggests that nonlinear mapping contributes to passive interaction force estimation, whereas the further improvement obtained by the GRU indicates that explicitly incorporating temporal dependence is beneficial for describing the interaction forces generated during continuous rehabilitation movements. To complement the task and interface level comparison presented in Figure 7, detailed component wise prediction metrics for the three estimators are summarized in Table 3. RMSE, MAE, and R^2^ were evaluated separately for *F_x_*, *F_y_*, and *F_z_* at the upper-arm and forearm interfaces, providing a more detailed assessment of model performance across the six force outputs.

Task-dependent differences were also observed in the model performance. The estimation characteristics differed between eating and drinking, particularly at the forearm interface, where the drinking task exhibited larger interaction force magnitudes and more pronounced temporal variations. These differences indicate that prediction difficulty is influenced not only by the selected estimator but also by the rehabilitation task and the physical location of the human–robot interface. Nevertheless, the relative performance advantage of the GRU was retained across the two tasks.

To further examine participant independent generalization, the GRU performance for each held out participant in the fivefold leave-one-participant-out evaluation is summarized in Table 4. In each outer fold, all movement cycles from the corresponding participant were excluded from model development and were used only for independent testing. The reported results therefore provide a direct assessment of passive interaction force estimation for participants who were not represented during model training.

The fold-wise results in Table 4 make the between participant variation in model performance explicit. Prediction accuracy was not identical across the five held-out participants, indicating that subject-specific biomechanical characteristics remain relevant to passive interaction force estimation. The variation among the outer folds, therefore, provides an estimate of the uncertainty associated with cross participant generalization in the present small cohort. These results should be interpreted as preliminary participant-independent validation rather than evidence of population level generalization.

At the component level, prediction accuracy remained dependent on the force direction and physical interface, as illustrated in Figure 6. At the upper-arm interface, the GRU achieved RMSE values of 0.96, 0.83, and 0.91 N for *F_x_*, *F_y_*, and *F_z_*, respectively, with corresponding R^2^ values of 0.49, 0.84, and 0.92. At the forearm interface, the RMSE values were 5.02, 2.07, and 5.11 N, respectively, while the corresponding *R*^2^ values were 0.68, 0.70, and 0.67. The larger absolute errors observed for forearm *F_x_* and *F_z_* were associated with their substantially larger force ranges.

Another feature in Figure 6 is that the predicted curves are smoother than the measured signals. Some short term fluctuations and sharp local peaks were not fully reproduced by the GRU model. This difference is more obvious for upper-arm *F_x_* and forearm *F_x_*. However, the main purpose of the proposed model is to describe the expected passive interaction state rather than to reproduce every small fluctuation in the force sensor signal. A smoother prediction is, therefore, acceptable for the following participation estimation, as long as the main force pattern and movement related changes are retained.

Overall, the results in Figure 6 and Figure 7 demonstrate that the GRU estimator captures the principal temporal patterns of passive interaction forces during repeated rehabilitation movements and generally provides better prediction performance than the reference models under both eating and drinking tasks. Although estimation accuracy varies with force component, task, and physical interface, the GRU provides a continuous passive interaction reference at both the upper-arm and forearm interfaces. The estimated passive force ${\hat{F}}^{p}\left(t\right)$ is subsequently used as the reference for calculating the interaction force residual in Section 3.2.

### 4.3. Controller-Level Simulation Protocol for Assistance Regulation

**Figure 8:**
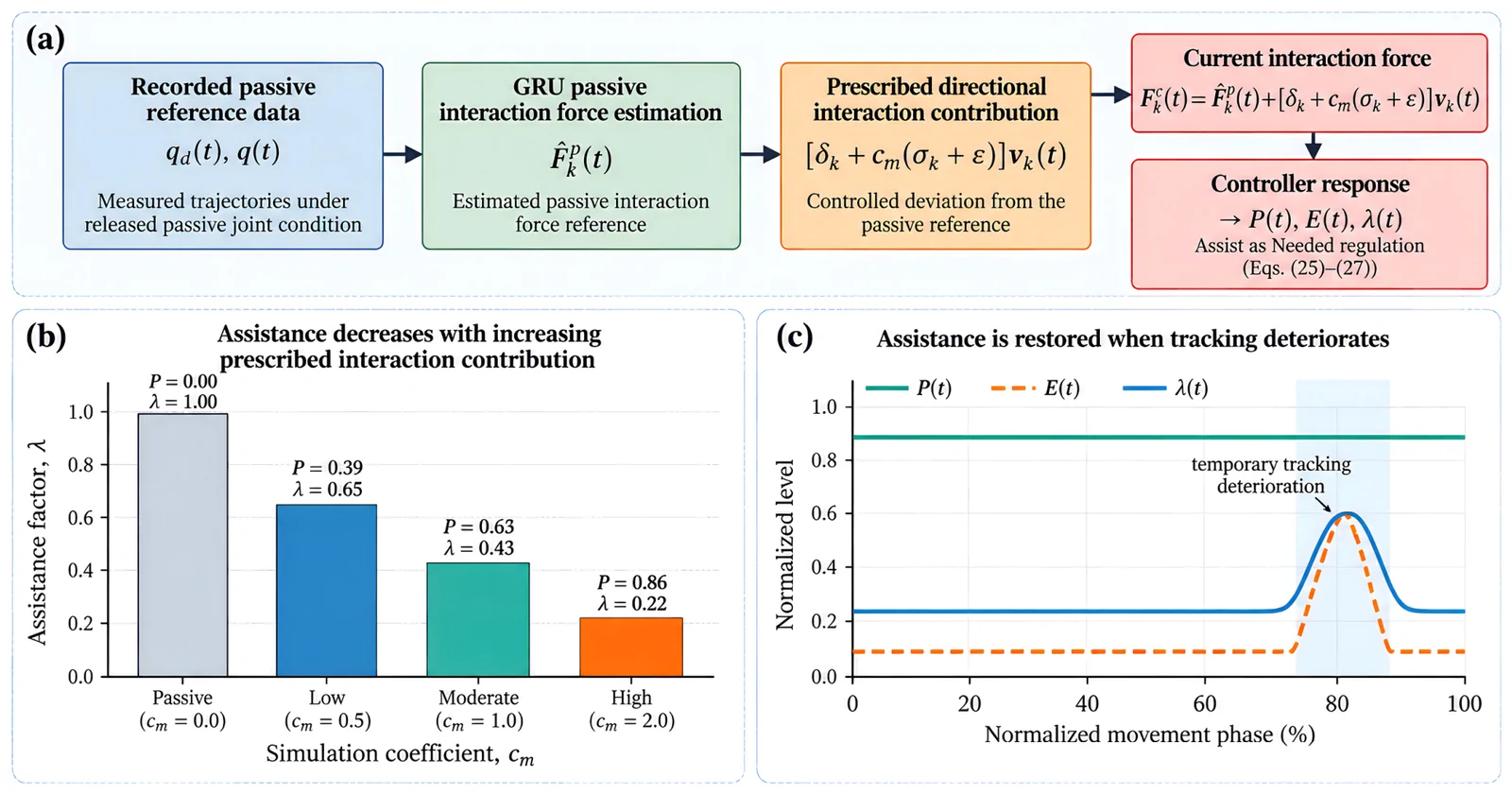
Controller-level simulation protocol for evaluation of the assistance regulation. (**a**) Flow diagram of the control framework, including passive interaction force estimation, prescribed interaction contribution calculation, and assist-as-needed controller response; (**b**) Assistance factor values under different levels of prescribed interaction contribution; (**c**) Dynamic response of the assistance regulation mechanism when tracking performance temporarily deteriorates.

Since active human subject experiments were not available in the present study, the proposed Assist-as-Needed strategy was evaluated through controller-level simulation. The purpose of this simulation was limited to examining the internal response of the control law to prescribed changes in directional interaction contribution and trajectory tracking performance. The simulation was not intended to reproduce human neuromuscular behavior or to validate the physiological meaning of the estimated participation level. Importantly, the present simulation does not incorporate a closed loop biomechanical or neuromusculoskeletal model of the human upper limb. The directional interaction contribution is prescribed externally through the simulation coefficient *c_m_*, rather than being generated dynamically from muscle activation, limb impedance, human adaptation, or changes in human motion caused by robotic assistance. Consequently, the simulation evaluates the internal response of the proposed controller but does not reproduce the bidirectional dynamic coupling between the human user and the rehabilitation robot. Accordingly, the simulated contribution levels should be interpreted as controlled interaction inputs used for proof of concept controller evaluation rather than experimentally measured levels of voluntary human effort. The measured trajectories obtained under the released passive joint condition and the passive interaction forces estimated by the GRU model were used as the basis of the simulation. The complete simulation procedure is illustrated in Figure 8.

For each physical interface *k* ∈ {*u*, *f*}, the GRU estimated force ${\hat{F}}^{p}\left(t\right)$ was taken as the expected passive interaction reference. A prescribed directional interaction component was then added along the desired movement direction to represent a controlled deviation from the passive interaction reference. The simulated current interaction force was defined as

(30) $$F_{k}^{c}(t)={\hat{F}}_{k}^{p}(t)+\left[\delta_{k}+c_{m}\left(\sigma_{k}+\varepsilon \right)\right]{\hat{v}}_{k}(t),$$

where *c_m_* is a dimensionless coefficient controlling the prescribed directional interaction contribution, *δ_k_* is the passive residual tolerance defined in Section 3.2, *σ_k_* is the corresponding passive residual scale, *ε* is the small positive constant used in Equation (18), and *v_k_*(*t*) denotes the unit vector along the desired movement direction. With this construction, the directional interaction component exceeding the passive uncertainty range can be adjusted systematically through *c_m_*, allowing the internal response of the controller to be evaluated under controlled conditions.

Four representative contribution conditions were considered,

*c_m_* ∈ {0, 0.5, 1.0, 2.0}, (31)

corresponding to passive, low, moderate, and high contribution conditions, respectively. These levels represent prescribed directional interaction contributions introduced for controller verification and should not be interpreted as quantified levels of voluntary human effort or clinical impairment. When *c_m_* = 0, no additional directional contribution was introduced and the simulated interaction state remained close to the passive condition. Increasing ***c_m_*** progressively increased the force component acting in the prescribed movement direction and therefore produced a higher estimated participation level through the procedure defined in Section 3.2.

In addition to user contribution, trajectory tracking performance was varied independently to evaluate the assistance regulation defined in Section 3.3. Under normal tracking, the normalized tracking error was maintained at *E*(*t*) = 0.10.

To examine whether the controller could restore assistance when movement performance deteriorated, a temporary tracking disturbance was introduced during the later part of the movement cycle. In this interval, *E*(*t*) was gradually increased from 0.10 to 0.60 and subsequently returned to its initial level. These values represent the normalized controller-level tracking error defined in Section 3.3 rather than direct joint angle errors; therefore, *E* = 0.10 and *E* = 0.60 correspond to different positions within the normalized tracking performance range bounded by *e*_0_ and *e*_1_. This test condition was designed to represent a case in which the user continued to contribute to the movement but temporarily failed to maintain the desired trajectory.

The participation estimate *P*(*t*) and tracking error *E*(*t*) were first used to calculate the raw assistance demand *λ*_raw_(*t*) according to Equation (25), after which the smoothed assistance factor *λ*(*t*) was obtained according to Equation (26). For the controller-level simulation, the assistance smoothing coefficient was maintained at *γ* = 0.90, as defined in Section 3.3.

The resulting assistance factor regulated the virtual stiffness and damping through Equation (27). A larger *λ*(*t*) therefore represented stronger robotic guidance, whereas a smaller value represented a more compliant interaction with reduced robotic assistance. The same simulation procedure was applied to the eating and drinking trajectories. Because the objective of this section was to evaluate the response of the control strategy rather than the task-specific biomechanics, the simulation variables were expressed in normalized form. This simulation design allows two key properties of the proposed controller to be examined independently. First, robotic assistance should decrease as the estimated user contribution increases while trajectory tracking remains acceptable. Second, assistance should increase again when tracking performance deteriorates, even when a relatively high user contribution is detected. The corresponding controller responses are evaluated in Section 4.4.

### 4.4. Controller Response Under Simulated Interaction Conditions

**Figure 9:**
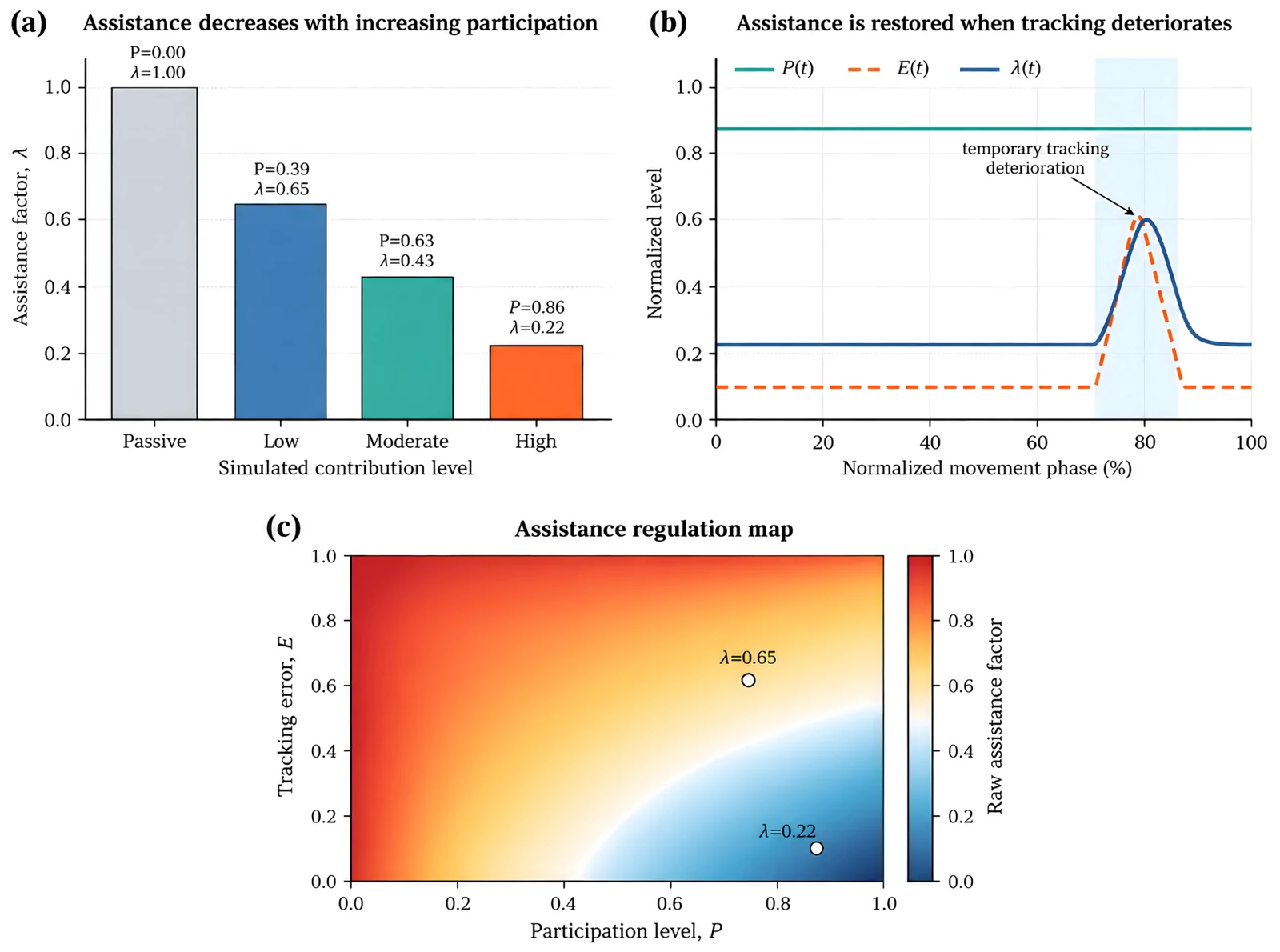
Assist-as-Needed controller response under simulated interaction conditions.

The simulated response of the proposed Assist-as-Needed controller is shown in Figure 9. The results first examine how robotic assistance changes with different levels of user contribution under normal trajectory tracking. The normalized tracking error was maintained at *E* = 0.10, while the simulated contribution coefficient was increased from the passive condition to the high contribution condition defined in Section 4.3.

As the estimated participation level increased, the required robotic assistance decreased progressively. Under the passive condition, *P* = 0 and the assistance factor remained at *λ* = 1. For the low, moderate, and high contribution conditions, the estimated participation levels were approximately 0.39, 0.63, and 0.86, respectively. According to the assistance regulation defined in Equation (25), the corresponding steady assistance factors decreased to approximately *λ* = 0.65, *λ* = 0.43, *λ* = 0.22, as shown in Figure 9a. Therefore, when trajectory tracking remained acceptable, an increase in user contribution directly reduced the amount of robotic intervention.

This behavior was also reflected in the adaptive impedance parameters. According to Equation (27), the normalized position of the stiffness and damping parameters within their allowable ranges is determined by *λ*(*t*). Under the high contribution condition, the virtual stiffness and damping were therefore reduced to approximately 22% of their available adaptive ranges above the predefined minimum values. The controller consequently became more compliant as the simulated user contribution increased. The second simulation examined whether robotic assistance could be restored when trajectory tracking deteriorated. The participation level was maintained at approximately *P* = 0.86, representing a high contribution condition. The tracking error remained at *E* = 0.10 during most of the movement cycle and was temporarily increased to 0.60 during the later phase of the movement. As illustrated in Figure 9b, the assistance factor was initially maintained at a low level of approximately 0.22. When the tracking error increased, the raw assistance demand increased to approximately *λ_raw_*= 0.65. After temporal smoothing, the assistance factor reached approximately 0.59 before returning toward its original low level when the tracking error recovered.

This result is important because the controller does not reduce assistance solely according to the estimated participation level. A high interaction contribution does not necessarily indicate that the desired movement is being performed correctly. When movement performance deteriorates, the tracking error term increases the assistance demand and allows the robot to provide additional trajectory guidance. In contrast, once tracking performance returns to the acceptable range, the additional assistance is gradually removed.

The overall relationship among participation, tracking performance, and robotic assistance is summarized in Figure 9c. When *P*(*t*) increases while *E*(*t*) remains small, the assistance factor decreases. In contrast, increasing *E*(*t*) shifts the controller toward a larger assistance level even when participation remains high. The proposed regulation, therefore, provides a continuous transition between user dominant and robot dominant rehabilitation rather than switching between fixed assistance modes.

These simulation results are consistent with the main principle of Assist-as-Needed rehabilitation, in which robotic support is reduced when the user contributes effectively and restored when movement performance becomes insufficient [15,16,17,18,19,20,21].

Similar concepts have been adopted in recent upper-limb rehabilitation controllers that adjust impedance or assistive force according to estimated motor capability and task performance [16,17,18,19,20,21,23,25,26,27,29,32].

It should be emphasized that the present results represent controller level-simulation rather than active human subject experiments. The simulation verifies only that the proposed control law responds in the intended direction when the prescribed interaction contribution and tracking performance are varied. It does not demonstrate that the estimated participation level is quantitatively correlated with actual voluntary human effort. Establishing such a relationship requires active human-in-the-loop experiments with independent measures of voluntary contribution. Therefore, the present results should be interpreted as proof of concept validation of the control logic rather than physiological validation of the participation estimator.

### 4.5. Discussion and Limitations

**Figure 10:**
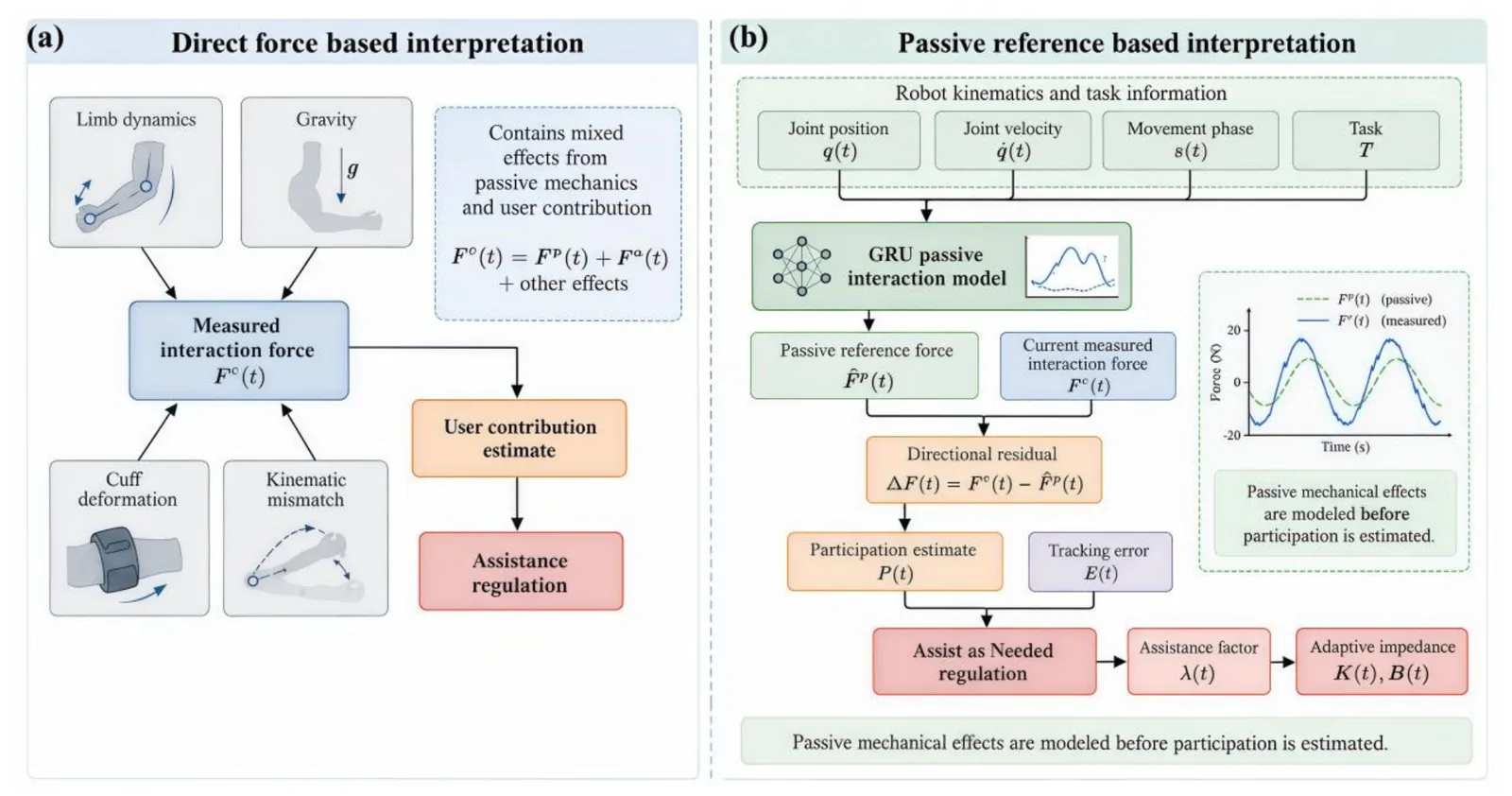
Conceptual comparison of direct force interpretation and passive reference-based assistance regulation.

The experimental and modeling results provide several observations relevant to interaction based Assist-as-Needed control. First, passive interaction was not invariant across the rehabilitation movement. Its magnitude and direction changed with task, physical interface, and movement phase, as demonstrated in Section 4.1 and Section 4.2. Consequently, the same absolute interaction force can correspond to different mechanical states under different movement conditions. This finding supports the use of a movement-dependent interaction reference when interpreting force changes during rehabilitation. The practical distinction between direct force interpretation and the proposed reference based strategy is summarized in Figure 10.

As illustrated in Figure 10a, direct force based regulation treats the measured interaction force as the main indicator of user contribution. However, the measured signal also contains passive effects caused by limb dynamics, gravity, cuff deformation, and residual kinematic mismatch. These components may change during movement even when the user does not actively contribute. Therefore, a change in measured force alone does not necessarily indicate a change in voluntary participation.

In contrast, the proposed strategy first estimates the expected passive interaction state from robot kinematics, movement phase, and task information, as shown in Figure 10b. The current measured interaction force is then compared with the GRU-estimated passive reference. Only the directional residual beyond the passive uncertainty range is used to form the participation estimate *P*(*t*). The participation estimate is further combined with the tracking error *E*(*t*) before the assistance factor *λ*(*t*) is determined. This layered interpretation prevents task-dependent passive mechanical variations from directly driving assistance regulation.

Another important feature of the proposed method is the use of two physical interfaces. The results in Figure 4 and Figure 5 showed that the relative magnitudes of the interaction forces measured at the upper-arm and forearm interfaces differed between the two tasks. During eating, the upper arm showed a relatively larger interface force share, whereas the relative forearm force share became more evident during drinking. This indicates that the two interfaces provide complementary information rather than repeated measurements of the same interaction state. The use of both interfaces therefore provides a more complete description of the physical interaction during upper limb movement.

The simulation results also show why participation should not be used alone to determine robotic assistance. In Section 4.4, the assistance factor decreased from 1.00 to approximately 0.22 as the simulated contribution increased while tracking remained acceptable. When the tracking error was temporarily increased, the assistance factor increased again even though the participation estimate remained high. This response indicates that the controller does not simply reduce assistance when a larger interaction contribution is detected. Instead, assistance is reduced only when the user contribution is accompanied by acceptable movement performance.

The proposed method still has several limitations. First, the passive interaction dataset was obtained from only five healthy participants, and the resulting cohort remains insufficient to represent the full inter subject variability of upper-limb biomechanics. The fivefold leave-one-participant-out validation reduces the risk of overly optimistic performance estimates caused by sample level information leakage and provides a participant independent evaluation of the GRU estimator. Nevertheless, the small cohort size does not eliminate the possibility of model overfitting to biomechanical characteristics represented in the present dataset. Participant specific anthropometric characteristics, including upper-arm and forearm lengths, limb mass distribution, anatomical joint center alignment, soft tissue compliance, and attachment geometry, may influence the passive interaction forces generated under the same prescribed robot trajectory. In the present experiments, anthropometric dimensions were measured and used to adjust the robot configuration and the positions of the upper-arm and forearm interfaces before data collection; however, these anthropometric variables were not explicitly included as inputs to the GRU estimator. Consequently, part of the variation observed across the held out participants may reflect subject-dependent biomechanical differences that cannot be represented solely by robot joint kinematics, movement phase, and task information. The present participant independent results should therefore be regarded as preliminary evidence of cross participant generalization rather than population level validation. Future studies should include a larger and more anthropometrically diverse cohort and investigate whether explicit anthropometric descriptors or subject-specific calibration can further improve passive interaction force estimation. Second, and importantly, the proposed participation estimation and Assist-as-Needed regulation have so far been evaluated only through controller-level simulation rather than active human-in-the-loop experiments. The present simulation also does not incorporate a closed loop biomechanical or neuromusculoskeletal model of the human upper-limb. Instead, the directional interaction contribution is prescribed externally through *c_m_*, while trajectory tracking performance is varied independently. This design is sufficient for examining whether the internal control law responds in the intended direction, but it cannot reproduce the bidirectional interaction in which robotic assistance modifies human motion, muscle activation, limb impedance, and the subsequent interaction forces. Consequently, the present simulation cannot evaluate human adaptation, muscle co contraction, fatigue, compensatory strategies, or the closed loop stability of the coupled human–robot system. Therefore, the present simulation should be interpreted as proof of concept validation of the control logic rather than a complete human-in-the-loop validation. The estimated participation level should presently be regarded as a control-oriented interaction indicator whose physiological and clinical validity remains to be established. Third, only eating and drinking were considered in the current experiments. More activities of daily living are required to evaluate whether the passive interaction reference can be extended to more complex upper limb movements. Finally, the prediction accuracy of the GRU was not uniform among all force components. Larger errors were observed in force directions with wider ranges and stronger local fluctuations, and these errors may influence the interaction residual used for participation estimation.

Future work should therefore focus first on active human-in-the-loop validation. Healthy participants will be instructed to perform controlled levels of voluntary contribution during the same eating and drinking trajectories, allowing the estimated participation level to be compared with independent indicators such as surface electromyography, interaction force residuals, and movement performance. Subsequent patient studies will then be required to determine whether the relationship between the estimated participation level and voluntary motor effort remains valid across different levels of neurological impairment and whether subject-specific calibration of the passive reference, residual threshold, and impedance range is necessary. In parallel with active human validation, future work may also incorporate a subject-specific upper-limb biomechanical or neuromusculoskeletal model into the controller loop to investigate the dynamic coupling and closed loop stability of the proposed assistance regulation under more realistic human–robot interaction conditions.

Overall, the present results indicate that incorporating a movement-dependent passive interaction estimate provides additional context for interpreting force changes before robotic assistance is adjusted. Together with trajectory tracking information, this enables assistance regulation to account for both the interaction state and movement performance. As summarized in Figure 10, the proposed framework first accounts for passive mechanical effects and then uses the remaining interaction change to estimate participation. This establishes a direct link between passive experimental data, participation estimation, and adaptive impedance regulation, and provides a methodological basis for subsequent active human validation of both the participation estimator and the adaptive assistance strategy.

## 5. Conclusions

This study developed an interaction force based Assist-as-Needed framework for a four DOF upper-limb rehabilitation robot. The framework combines a learned passive interaction force estimator, directional residual based participation estimation, trajectory tracking evaluation, and adaptive impedance regulation within a unified control architecture. Passive rehabilitation experiments during eating and drinking showed that the human–robot interaction force was affected by rehabilitation task, physical interface, and force direction. Releasing the passive joints generally reduced the interaction force compared with the locked condition, while the relative interaction force pattern between the upper-arm and forearm interfaces changed with the rehabilitation task. These results confirmed that passive interaction cannot be represented by a single constant force and supported the use of task and interface information in the passive interaction model.

A GRU model was then used to estimate the three dimensional passive interaction forces at the upper-arm and forearm interfaces. The estimated forces reproduced the main temporal patterns of the measured signals over the movement cycle. The best agreement was obtained for the upper-arm *F_z_*, with an R^2^ of approximately 0.92, while the prediction performance varied among different force components. The resulting passive force estimate was subsequently used to calculate directional interaction residuals and derive a normalized participation variable.

The Assist-as-Needed controller combined the estimated participation level with trajectory tracking performance to regulate the assistance factor and robot impedance. Controller-level simulation showed that the assistance factor decreased from 1.00 to approximately 0.22 as the simulated contribution increased from the passive to the high contribution condition while tracking remained acceptable. When the tracking error was temporarily increased, the assistance factor increased again, showing that robotic support could be restored when movement performance deteriorated even under a high participation condition.

Overall, the present results support the feasibility of using a learned passive interaction reference within an interaction force based Assist-as-Needed control framework. The proposed method links passive experimental data, a control-oriented participation estimate, and adaptive impedance regulation within a unified controller. However, the active assistance results were obtained only through controller-level simulation. Therefore, the present study demonstrates the internal consistency and expected response of the control strategy but does not yet validate the estimated participation level as a quantitative measure of voluntary human effort. Active human experiments, subsequent patient studies, and more realistic biomechanical human-in-the-loop modeling are required to establish this relationship and further evaluate the physiological validity and closed loop performance of the proposed framework.

## Figures and Tables

**Table 1:** GRU architecture and training settings.

Category	Setting	Category	Setting	Category	Setting
GRU layers	2	Hidden units	64/32	Input/output dimensions	11/6
Sequence length	50 samples	Dropout rate	0.20	Loss function	MSE
Optimizer	Adam	Initial learning rate	1 × 10^−3^	Batch size	32
Maximum epochs	200	Early stopping patience	20 epochs	*L*_2_ weight decay	1 × 10^−5^
Cross-validation	5 fold LOSO	Training/validation split	80%/20% by cycle	Sampling frequency	100 Hz

**Table 2:** Summary of resultant passive interaction forces under different rehabilitation conditions.

Task	Interface	Passive Joint Condition	Resultant Force *F_total_* (N)
Eating	Forearm	Released	2.69 ± 0.72
Eating	Forearm	Locked	4.39 ± 1.10
Eating	Upper arm	Released	3.18 ± 0.83
Eating	Upper arm	Locked	5.12 ± 1.28
Drinking	Forearm	Released	5.33 ± 1.21
Drinking	Forearm	Locked	7.85 ± 1.57
Drinking	Upper arm	Released	3.64 ± 0.90
Drinking	Upper arm	Locked	4.14 ± 1.01

**Table 3:** Component-wise prediction performance comparison among LR, MLP, and GRU estimators.

Interface	Force Component	LR	MLP	GRU
Upper arm	*F_x_*	1.18/0.91/0.23	1.04/0.80/0.40	0.96/0.73/0.49
Upper arm	*F_y_*	1.09/0.84/0.72	0.94/0.72/0.79	0.83/0.63/0.84
Upper arm	*F_z_*	1.22/0.94/0.86	1.06/0.81/0.89	0.91/0.69/0.92
Forearm	*F_x_*	6.42/4.97/0.48	5.63/4.33/0.60	5.02/3.84/0.68
Forearm	*F_y_*	2.71/2.08/0.49	2.34/1.79/0.62	2.07/1.58/0.70
Forearm	*F_z_*	6.61/5.10/0.45	5.76/4.43/0.58	5.11/3.91/0.67

**Table 4:** Participant independent performance of the GRU estimator under fivefold leave-one-participant-out cross-validation.

Held-Out Participant	Upper Arm RMSE (N)	Upper Arm MAE (N)	Upper Arm \(R^2^\)	Forearm RMSE (N)	Forearm MAE (N)	Forearm\(R^2^\)
P1	0.84	0.64	0.77	3.82	2.94	0.70
P2	0.91	0.69	0.74	4.11	3.14	0.68
P3	0.88	0.67	0.76	3.96	3.04	0.69
P4	0.95	0.72	0.72	4.28	3.27	0.65
P5	0.92	0.70	0.75	4.17	3.18	0.67
**Mean ± SD**	**0.90 ± 0.04**	**0.68 ± 0.03**	**0.75 ± 0.02**	**4.07 ± 0.18**	**3.11 ± 0.13**	**0.68 ± 0.02**

Notes: For each held-out participant, RMSE, MAE, and R^2^ were first calculated for the individual Cartesian force components under the two rehabilitation tasks and then summarized within each physical interface.

## Data Availability

The data presented in this study are available on request from the corresponding author.
